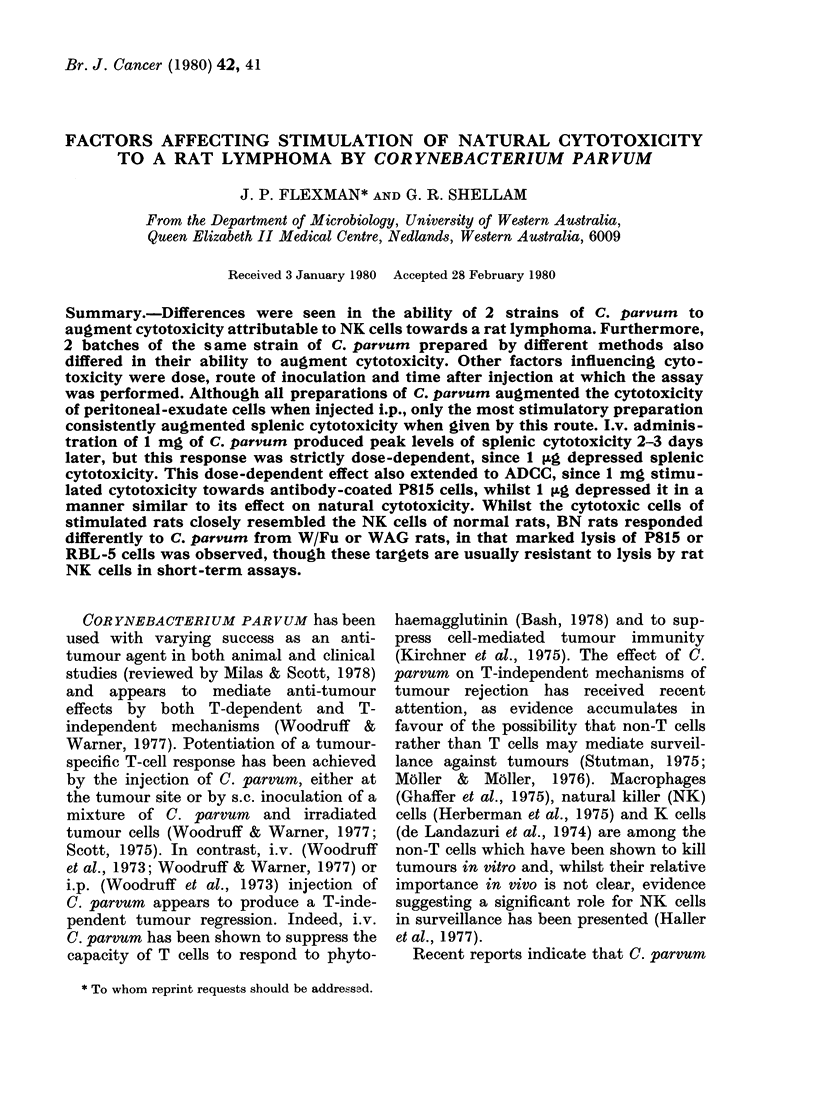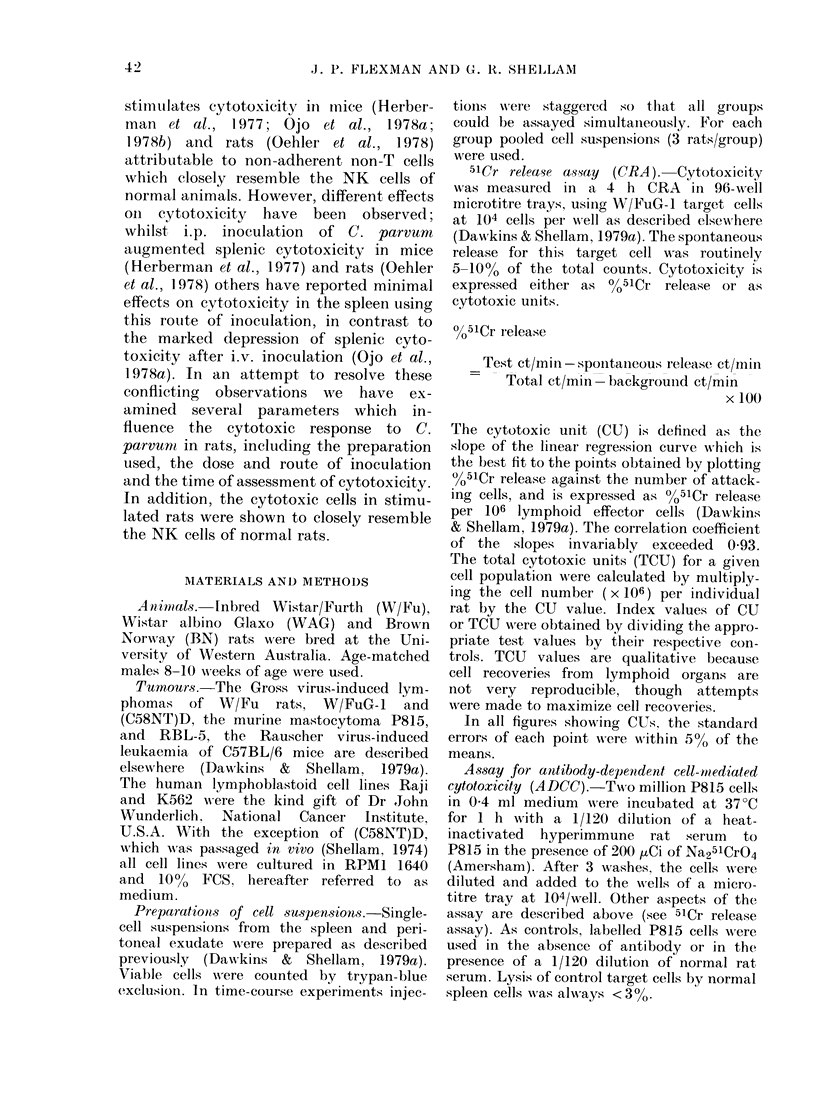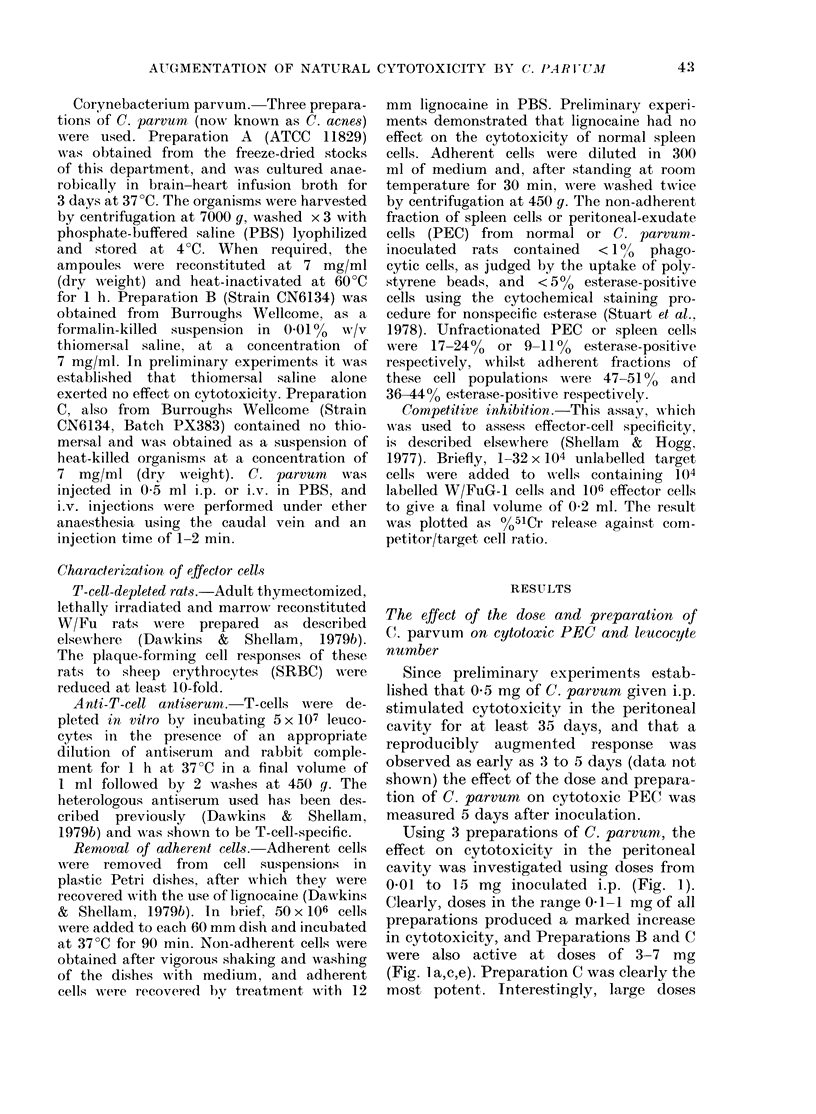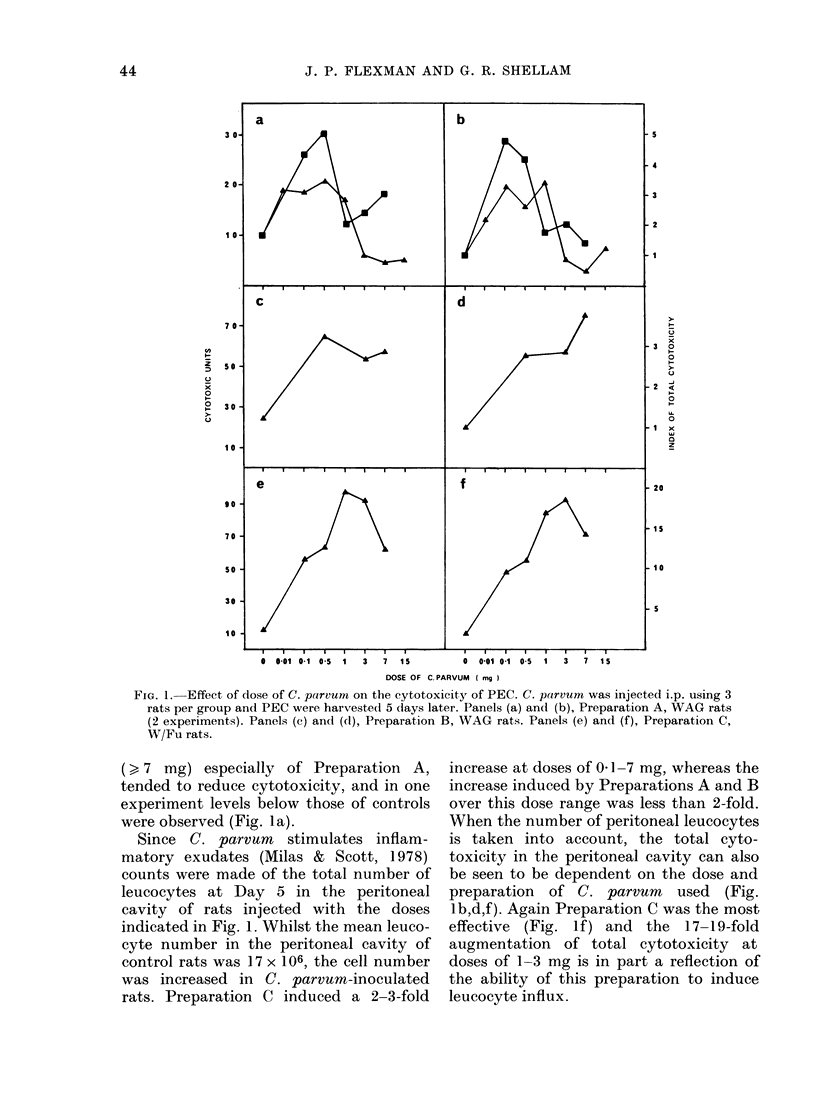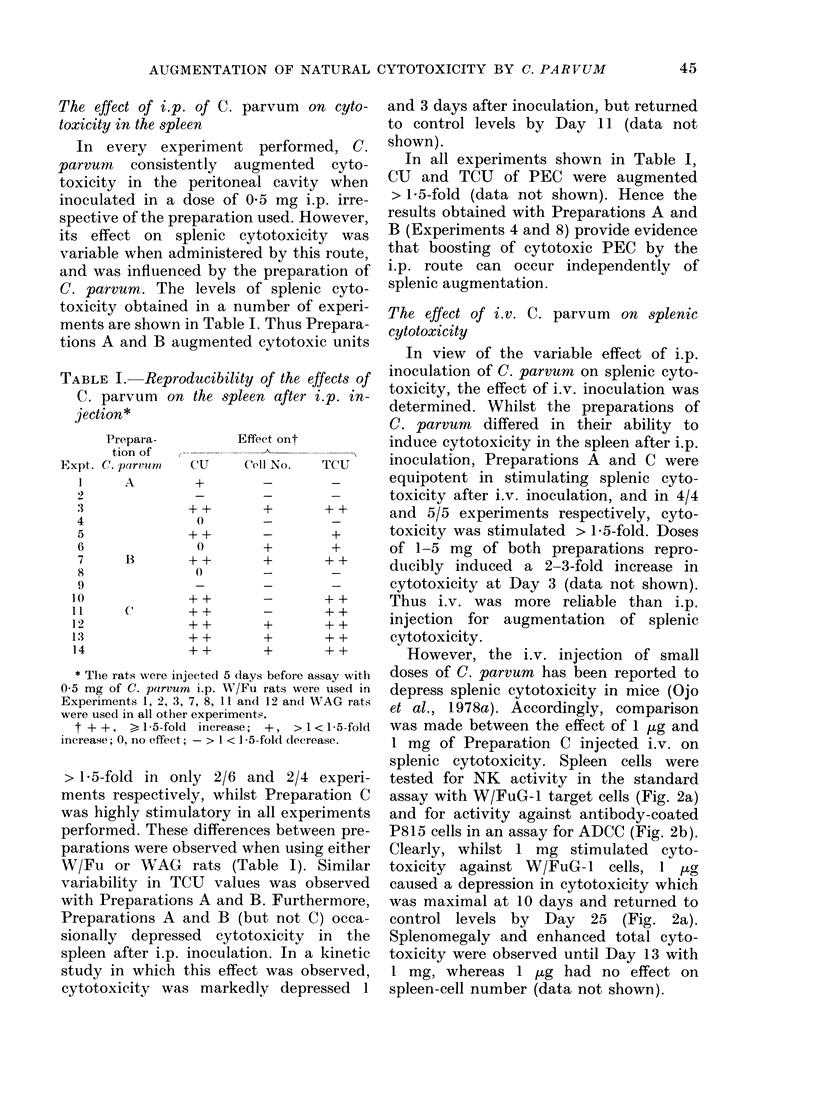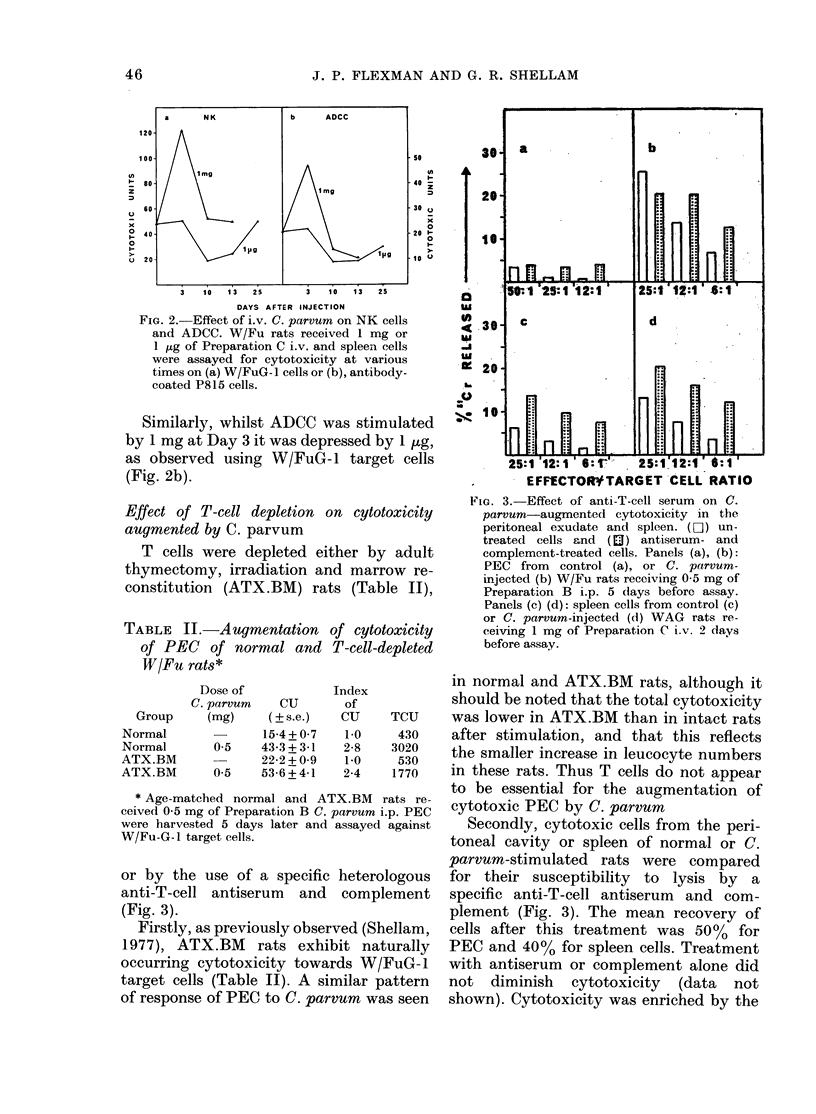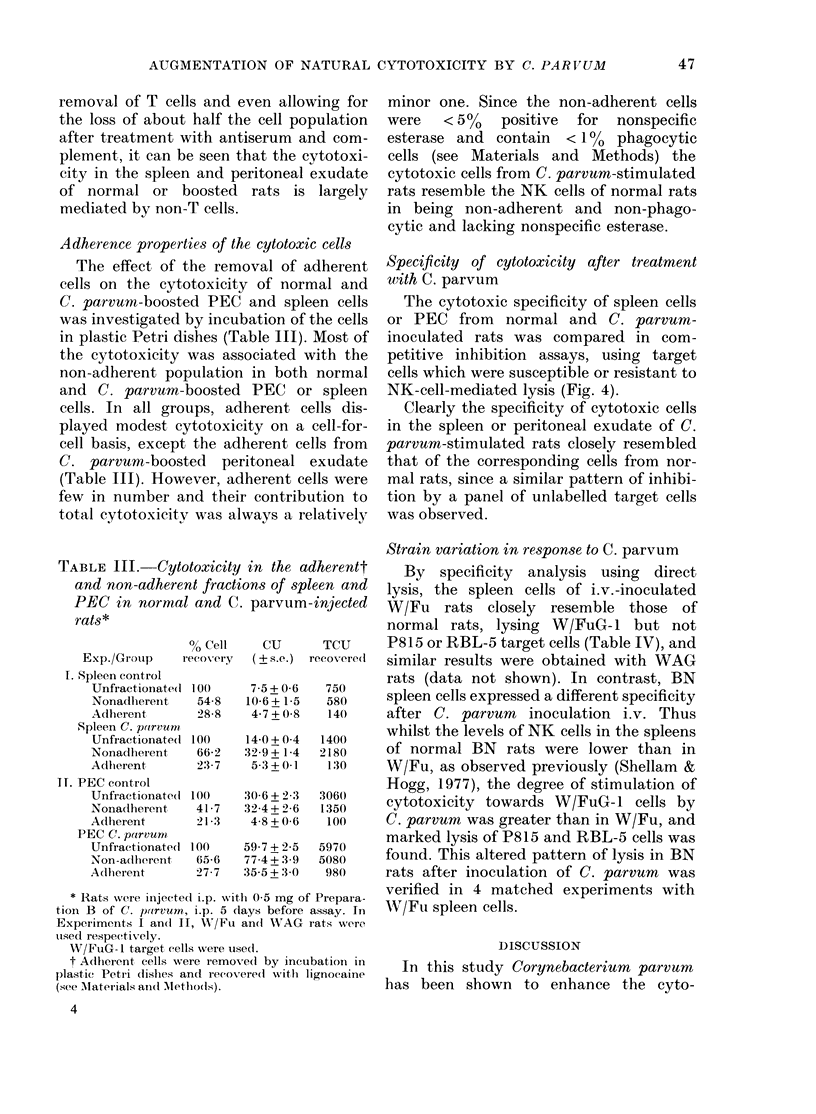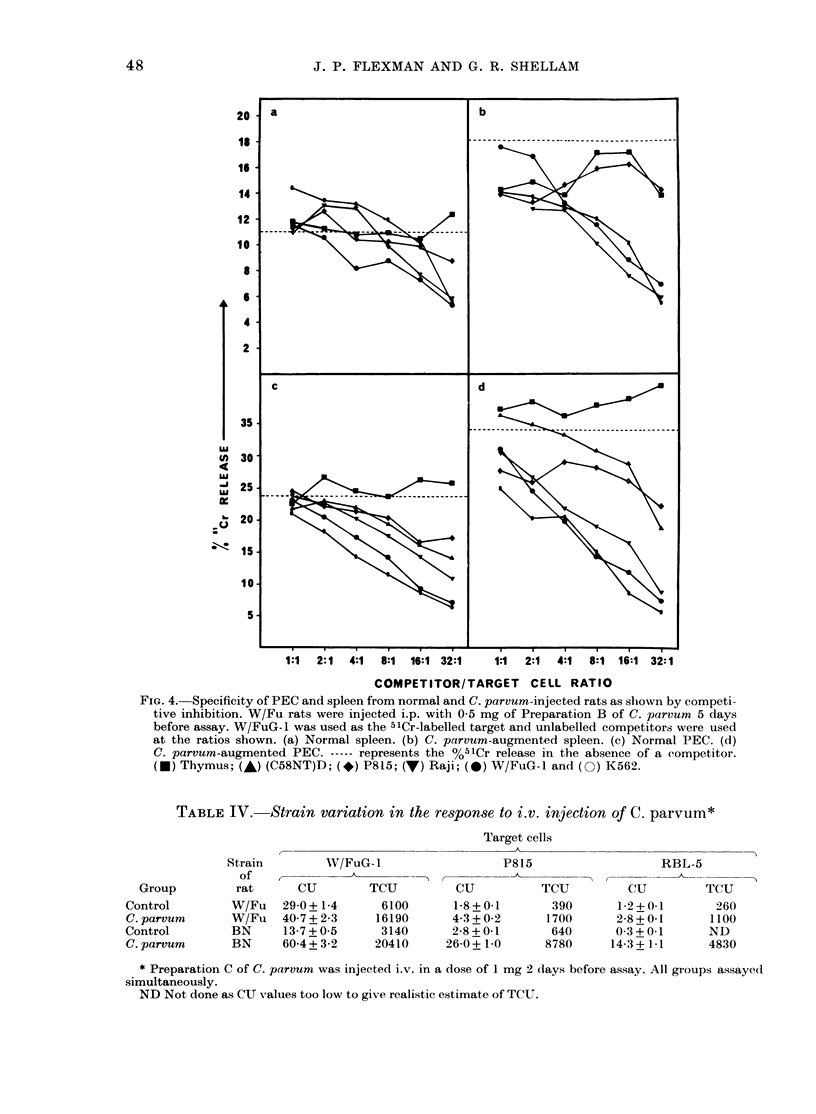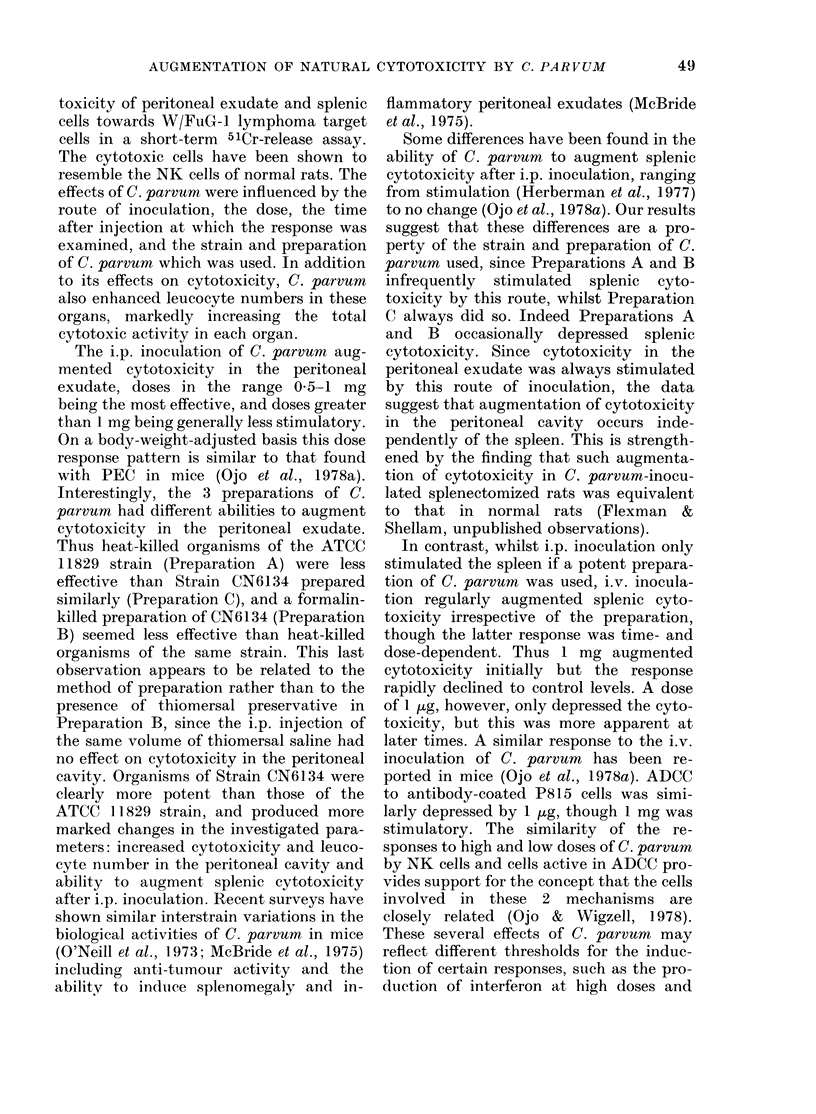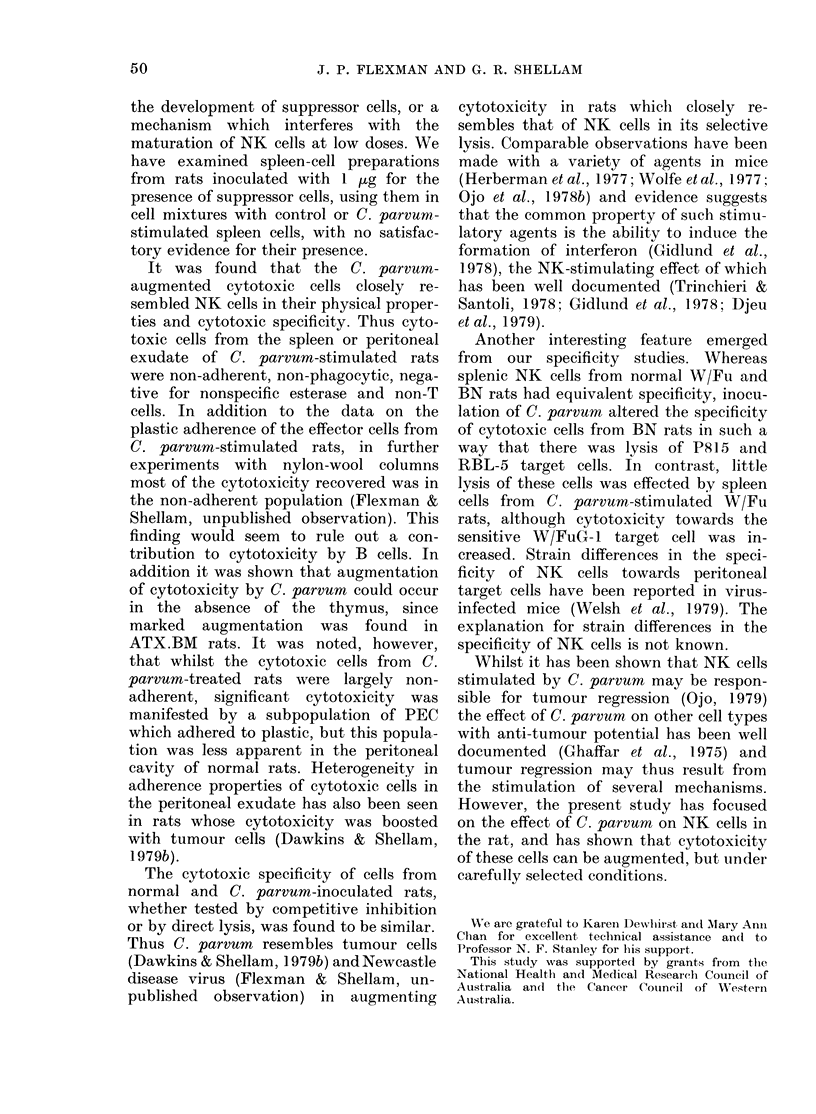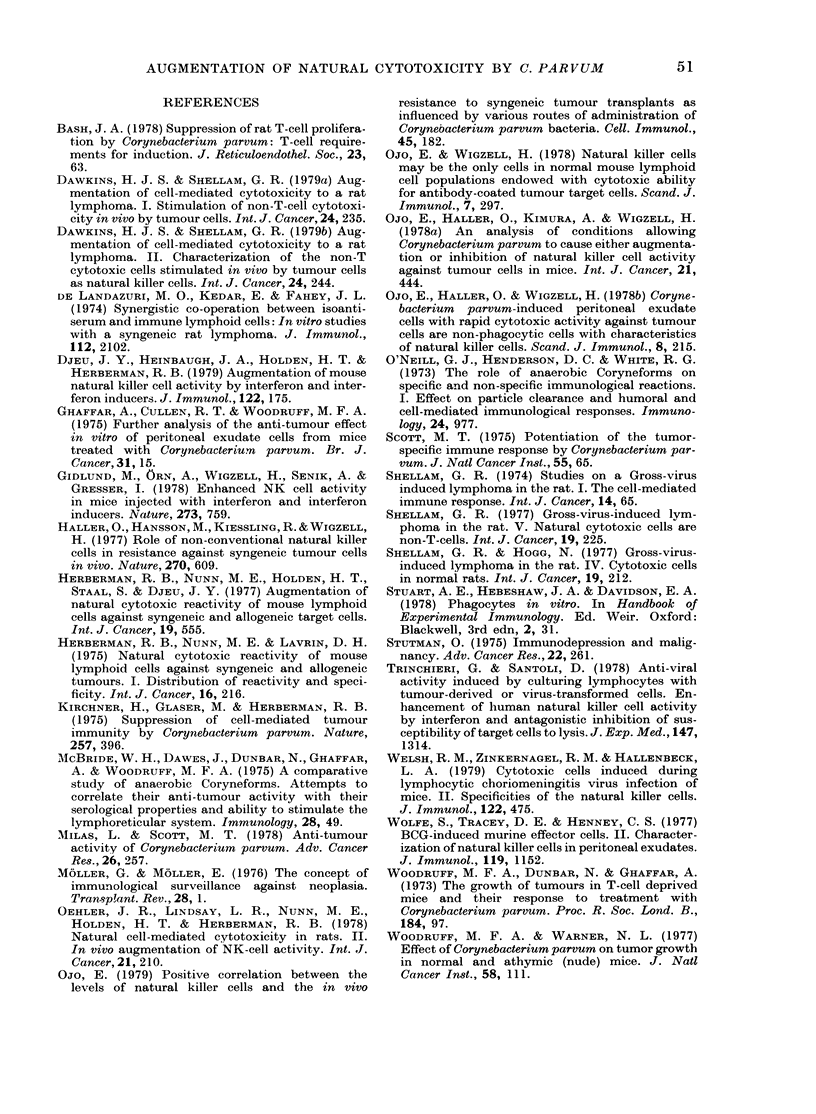# Factors affecting stimulation of natural cytotoxicity to a rat lymphoma by Corynebacterium parvum.

**DOI:** 10.1038/bjc.1980.201

**Published:** 1980-07

**Authors:** J. P. Flexman, G. R. Shellam

## Abstract

Differences were seen in the ability of 2 strains of C. parvum to augment cytotoxicity attributable to NK cells towards a rat lymphoma. Furthermore, 2 batches of the same strain of C. parvum prepared by different methods also differed in their ability to augment cytotoxicity. Other factors influencing cytotoxicity were dose, route of inoculation and time after injection at which the assay was performed. Although all preparations of C. parvum augmented the cytotoxicity of peritoneal-exudate cells when injected i.p., only the most stimulatory preparation consistently augmented splenic cytotoxicity when given by this route. I.v. administration of 1 mg of C. parvum produced peak levels of splenic cytotoxicity 2-3 days later, but this response was strictly dose-dependent, since 1 microgram depressed splenic cytotoxicity. This dose-dependent effect also extended to ADCC, since 1 mg stimulated cytotoxicity towards antibody-coated P815 cells, whilst 1 microgram depressed it in a manner similar to its effect on natural cytotoxicity. Whilst the cytotoxic cells of stimulated rats closely resembled the NK cells of normal rats, BN rats responded differently to C. parvum from W/Fu or WAG rats, in that marked lysis of P815 or RBL-5 cells was observed, though these targets are usually resistant to lysis by rat NK cells in short-term assays.


*Br. J. Cancer* (1980) **42**, 41

### FACTORS AFFECTING STIMULATION OF NATURAL CYTOTOXICITY TO A RAT LYMPHOMA BY *CORYNEBACTERIUM PARVUM*

J. P. FLEXMAN* AND G. R. SHELLAM

*From the Department of Microbiology, University of Western Australia, Queen Elizabeth II Medical Centre, Nedlands, Western Australia, 6009*

Received 3 January 1980 Accepted 28 February 1980

**Summary.—Differences were seen in the ability of 2 strains of *C. parvum* to augment cytotoxicity attributable to NK cells towards a rat lymphoma. Furthermore, 2 batches of the same strain of *C. parvum* prepared by different methods also differed in their ability to augment cytotoxicity. Other factors influencing cytotoxicity were dose, route of inoculation and time after injection at which the assay was performed. Although all preparations of *C. parvum* augmented the cytotoxicity of peritoneal-exudate cells when injected i.p., only the most stimulatory preparation consistently augmented splenic cytotoxicity when given by this route. I.v. administration of 1 mg of *C. parvum* produced peak levels of splenic cytotoxicity 2–3 days later, but this response was strictly dose-dependent, since 1 μg depressed splenic cytotoxicity. This dose-dependent effect also extended to ADCC, since 1 mg stimulated cytotoxicity towards antibody-coated P815 cells, whilst 1 μg depressed it in a manner similar to its effect on natural cytotoxicity. Whilst the cytotoxic cells of stimulated rats closely resembled the NK cells of normal rats, BN rats responded differently to *C. parvum* from W/Fu or WAG rats, in that marked lysis of P815 or RBL-5 cells was observed, though these targets are usually resistant to lysis by rat NK cells in short-term assays.**

CORYNEBACTERIUM PARVUM has been used with varying success as an anti-tumour agent in both animal and clinical studies (reviewed by Milas & Scott, 1978) and appears to mediate anti-tumour effects by both T-dependent and T-independent mechanisms (Woodruff & Warner, 1977). Potentiation of a tumour-specific T-cell response has been achieved by the injection of *C. parvum*, either at the tumour site or by s.c. inoculation of a mixture of *C. parvum* and irradiated tumour cells (Woodruff & Warner, 1977; Scott, 1975). In contrast, i.v. (Woodruff *et al.*, 1973; Woodruff & Warner, 1977) or i.p. (Woodruff *et al.*, 1973) injection of *C. parvum* appears to produce a T-independent tumour regression. Indeed, i.v. *C. parvum* has been shown to suppress the capacity of T cells to respond to phytohaemagglutinin (Bash, 1978) and to suppress cell-mediated tumour immunity (Kirchner *et al.*, 1975). The effect of *C. parvum* on T-independent mechanisms of tumour rejection has received recent attention, as evidence accumulates in favour of the possibility that non-T cells rather than T cells may mediate surveillance against tumours (Stutman, 1975; Möller & Möller, 1976). Macrophages (Ghaffer *et al.*, 1975), natural killer (NK) cells (Herberman *et al.*, 1975) and K cells (de Landazuri *et al.*, 1974) are among the non-T cells which have been shown to kill tumours *in vitro* and, whilst their relative importance *in vivo* is not clear, evidence suggesting a significant role for NK cells in surveillance has been presented (Haller *et al.*, 1977).

Recent reports indicate that *C. parvum*

* To whom reprint requests should be addressed.

stimulates cytotoxicity in mice (Herberman *et al.*, 1977; Ojo *et al.*, 1978*a*; 1978*b*) and rats (Oehler *et al.*, 1978) attributable to non-adherent non-T cells which closely resemble the NK cells of normal animals. However, different effects on cytotoxicity have been observed; whilst i.p. inoculation of *C. parvum* augmented splenic cytotoxicity in mice (Herberman *et al.*, 1977) and rats (Oehler *et al.*, 1978) others have reported minimal effects on cytotoxicity in the spleen using this route of inoculation, in contrast to the marked depression of splenic cytotoxicity after i.v. inoculation (Ojo *et al.*, 1978*a*). In an attempt to resolve these conflicting observations we have examined several parameters which influence the cytotoxic response to *C. parvum* in rats, including the preparation used, the dose and route of inoculation and the time of assessment of cytotoxicity. In addition, the cytotoxic cells in stimulated rats were shown to closely resemble the NK cells of normal rats.

### MATERIALS AND METHODS

*Animals.*—Inbred Wistar/Furth (W/Fu), Wistar albino Glaxo (WAG) and Brown Norway (BN) rats were bred at the University of Western Australia. Age-matched males 8–10 weeks of age were used.

*Tumours.*—The Gross virus-induced lymphomas of W/Fu rats, W/FuG-1 and (C58NT)D, the murine mastocytoma P815, and RBL-5, the Rauscher virus-induced leukaemia of C57BL/6 mice are described elsewhere (Dawkins & Shellam, 1979*a*). The human lymphoblastoid cell lines Raji and K562 were the kind gift of Dr John Wunderlich, National Cancer Institute, U.S.A. With the exception of (C58NT)D, which was passaged *in vivo* (Shellam, 1974) all cell lines were cultured in RPMI 1640 and 10% FCS, hereafter referred to as medium.

*Preparations of cell suspensions.*—Single-cell suspensions from the spleen and peritoneal exudate were prepared as described previously (Dawkins & Shellam, 1979*a*). Viable cells were counted by trypan-blue exclusion. In time-course experiments injections were staggered so that all groups could be assayed simultaneously. For each group pooled cell suspensions (3 rats/group) were used.

*$^{51}$Cr release assay* (*CRA*).—Cytotoxicity was measured in a 4 h CRA in 96-well microtitre trays, using W/FuG-1 target cells at $10^4$ cells per well as described elsewhere (Dawkins & Shellam, 1979*a*). The spontaneous release for this target cell was routinely 5–10% of the total counts. Cytotoxicity is expressed either as %$^{51}$Cr release or as cytotoxic units.

$$\%^{51}\text{Cr release} = \frac{\text{Test ct/min} - \text{spontaneous release ct/min}}{\text{Total ct/min} - \text{background ct/min}} \times 100$$

The cytotoxic unit (CU) is defined as the slope of the linear regression curve which is the best fit to the points obtained by plotting %$^{51}$Cr release against the number of attacking cells, and is expressed as %$^{51}$Cr release per $10^6$ lymphoid effector cells (Dawkins & Shellam, 1979*a*). The correlation coefficient of the slopes invariably exceeded 0·93. The total cytotoxic units (TCU) for a given cell population were calculated by multiplying the cell number ($\times 10^6$) per individual rat by the CU value. Index values of CU or TCU were obtained by dividing the appropriate test values by their respective controls. TCU values are qualitative because cell recoveries from lymphoid organs are not very reproducible, though attempts were made to maximize cell recoveries.

In all figures showing CUs, the standard errors of each point were within 5% of the means.

*Assay for antibody-dependent cell-mediated cytotoxicity* (*ADCC*).—Two million P815 cells in 0·4 ml medium were incubated at 37 °C for 1 h with a 1/120 dilution of a heat-inactivated hyperimmune rat serum to P815 in the presence of 200 $\mu$Ci of $Na_2{}^{51}CrO_4$ (Amersham). After 3 washes, the cells were diluted and added to the wells of a microtitre tray at $10^4$/well. Other aspects of the assay are described above (see $^{51}$Cr release assay). As controls, labelled P815 cells were used in the absence of antibody or in the presence of a 1/120 dilution of normal rat serum. Lysis of control target cells by normal spleen cells was always $<3\%$.

Corynebacterium parvum.—Three preparations of *C. parvum* (now known as *C. acnes*) were used. Preparation A (ATCC 11829) was obtained from the freeze-dried stocks of this department, and was cultured anaerobically in brain–heart infusion broth for 3 days at 37°C. The organisms were harvested by centrifugation at 7000 *g*, washed $\times 3$ with phosphate-buffered saline (PBS) lyophilized and stored at 4°C. When required, the ampoules were reconstituted at 7 mg/ml (dry weight) and heat-inactivated at 60°C for 1 h. Preparation B (Strain CN6134) was obtained from Burroughs Wellcome, as a formalin-killed suspension in 0·01% w/v thiomersal saline, at a concentration of 7 mg/ml. In preliminary experiments it was established that thiomersal saline alone exerted no effect on cytotoxicity. Preparation C, also from Burroughs Wellcome (Strain CN6134, Batch PX383) contained no thiomersal and was obtained as a suspension of heat-killed organisms at a concentration of 7 mg/ml (dry weight). *C. parvum* was injected in 0·5 ml i.p. or i.v. in PBS, and i.v. injections were performed under ether anaesthesia using the caudal vein and an injection time of 1–2 min.

*Characterization of effector cells*

*T-cell-depleted rats.*—Adult thymectomized, lethally irradiated and marrow reconstituted W/Fu rats were prepared as described elsewhere (Dawkins & Shellam, 1979*b*). The plaque-forming cell responses of these rats to sheep erythrocytes (SRBC) were reduced at least 10-fold.

*Anti-T-cell antiserum.*—T-cells were depleted *in vitro* by incubating $5 \times 10^7$ leucocytes in the presence of an appropriate dilution of antiserum and rabbit complement for 1 h at 37°C in a final volume of 1 ml followed by 2 washes at 450 *g*. The heterologous antiserum used has been described previously (Dawkins & Shellam, 1979*b*) and was shown to be T-cell-specific.

*Removal of adherent cells.*—Adherent cells were removed from cell suspensions in plastic Petri dishes, after which they were recovered with the use of lignocaine (Dawkins & Shellam, 1979*b*). In brief, $50 \times 10^6$ cells were added to each 60 mm dish and incubated at 37°C for 90 min. Non-adherent cells were obtained after vigorous shaking and washing of the dishes with medium, and adherent cells were recovered by treatment with 12 mm lignocaine in PBS. Preliminary experiments demonstrated that lignocaine had no effect on the cytotoxicity of normal spleen cells. Adherent cells were diluted in 300 ml of medium and, after standing at room temperature for 30 min, were washed twice by centrifugation at 450 *g*. The non-adherent fraction of spleen cells or peritoneal-exudate cells (PEC) from normal or *C. parvum*-inoculated rats contained $<1\%$ phagocytic cells, as judged by the uptake of polystyrene beads, and $<5\%$ esterase-positive cells using the cytochemical staining procedure for nonspecific esterase (Stuart *et al.*, 1978). Unfractionated PEC or spleen cells were 17–24% or 9–11% esterase-positive respectively, whilst adherent fractions of these cell populations were 47–51% and 36–44% esterase-positive respectively.

*Competitive inhibition.*—This assay, which was used to assess effector-cell specificity, is described elsewhere (Shellam & Hogg, 1977). Briefly, $1\text{–}32 \times 10^4$ unlabelled target cells were added to wells containing $10^4$ labelled W/FuG-1 cells and $10^6$ effector cells to give a final volume of 0·2 ml. The result was plotted as $\%^{51}$Cr release against competitor/target cell ratio.

RESULTS

*The effect of the dose and preparation of* C. parvum *on cytotoxic PEC and leucocyte number*

Since preliminary experiments established that 0·5 mg of *C. parvum* given i.p. stimulated cytotoxicity in the peritoneal cavity for at least 35 days, and that a reproducibly augmented response was observed as early as 3 to 5 days (data not shown) the effect of the dose and preparation of *C. parvum* on cytotoxic PEC was measured 5 days after inoculation.

Using 3 preparations of *C. parvum*, the effect on cytotoxicity in the peritoneal cavity was investigated using doses from 0·01 to 15 mg inoculated i.p. (Fig. 1). Clearly, doses in the range 0·1–1 mg of all preparations produced a marked increase in cytotoxicity, and Preparations B and C were also active at doses of 3–7 mg (Fig. 1a,c,e). Preparation C was clearly the most potent. Interestingly, large doses

FIG. 1.—Effect of dose of *C. parvum* on the cytotoxicity of PEC. *C. parvum* was injected i.p. using 3 rats per group and PEC were harvested 5 days later. Panels (a) and (b), Preparation A, WAG rats (2 experiments). Panels (c) and (d), Preparation B, WAG rats. Panels (e) and (f), Preparation C, W/Fu rats.

($\geqslant$7 mg) especially of Preparation A, tended to reduce cytotoxicity, and in one experiment levels below those of controls were observed (Fig. 1a).

Since *C. parvum* stimulates inflammatory exudates (Milas & Scott, 1978) counts were made of the total number of leucocytes at Day 5 in the peritoneal cavity of rats injected with the doses indicated in Fig. 1. Whilst the mean leucocyte number in the peritoneal cavity of control rats was $17 \times 10^6$, the cell number was increased in *C. parvum*-inoculated rats. Preparation C induced a 2–3-fold increase at doses of 0·1–7 mg, whereas the increase induced by Preparations A and B over this dose range was less than 2-fold. When the number of peritoneal leucocytes is taken into account, the total cytotoxicity in the peritoneal cavity can also be seen to be dependent on the dose and preparation of *C. parvum* used (Fig. 1b,d,f). Again Preparation C was the most effective (Fig. 1f) and the 17–19-fold augmentation of total cytotoxicity at doses of 1–3 mg is in part a reflection of the ability of this preparation to induce leucocyte influx.

*The effect of i.p. of* C. parvum *on cytotoxicity in the spleen*

In every experiment performed, *C. parvum* consistently augmented cytotoxicity in the peritoneal cavity when inoculated in a dose of 0·5 mg i.p. irrespective of the preparation used. However, its effect on splenic cytotoxicity was variable when administered by this route, and was influenced by the preparation of *C. parvum*. The levels of splenic cytotoxicity obtained in a number of experiments are shown in Table I. Thus Preparations A and B augmented cytotoxic units

TABLE I.—*Reproducibility of the effects of* C. parvum *on the spleen after i.p. injection**

| Expt. | Preparation of *C. parvum* | Effect on† CU | Cell No. | TCU |
|---|---|---|---|---|
| 1 | A | + | – | – |
| 2 | | – | – | – |
| 3 | | + + | + | + + |
| 4 | | 0 | – | – |
| 5 | | + + | – | + |
| 6 | | 0 | + | + |
| 7 | B | + + | + | + + |
| 8 | | 0 | – | – |
| 9 | | – | – | – |
| 10 | | + + | – | + + |
| 11 | C | + + | – | + + |
| 12 | | + + | + | + + |
| 13 | | + + | + | + + |
| 14 | | + + | + | + + |

* The rats were injected 5 days before assay with 0·5 mg of *C. parvum* i.p. W/Fu rats were used in Experiments 1, 2, 3, 7, 8, 11 and 12 and WAG rats were used in all other experiments.

† + +, ⩾ 1·5-fold increase; +, > 1 < 1·5-fold increase; 0, no effect; – > 1 < 1·5-fold decrease.

> 1·5-fold in only 2/6 and 2/4 experiments respectively, whilst Preparation C was highly stimulatory in all experiments performed. These differences between preparations were observed when using either W/Fu or WAG rats (Table I). Similar variability in TCU values was observed with Preparations A and B. Furthermore, Preparations A and B (but not C) occasionally depressed cytotoxicity in the spleen after i.p. inoculation. In a kinetic study in which this effect was observed, cytotoxicity was markedly depressed 1 and 3 days after inoculation, but returned to control levels by Day 11 (data not shown).

In all experiments shown in Table I, CU and TCU of PEC were augmented > 1·5-fold (data not shown). Hence the results obtained with Preparations A and B (Experiments 4 and 8) provide evidence that boosting of cytotoxic PEC by the i.p. route can occur independently of splenic augmentation.

*The effect of i.v.* C. parvum *on splenic cytotoxicity*

In view of the variable effect of i.p. inoculation of *C. parvum* on splenic cytotoxicity, the effect of i.v. inoculation was determined. Whilst the preparations of *C. parvum* differed in their ability to induce cytotoxicity in the spleen after i.p. inoculation, Preparations A and C were equipotent in stimulating splenic cytotoxicity after i.v. inoculation, and in 4/4 and 5/5 experiments respectively, cytotoxicity was stimulated > 1·5-fold. Doses of 1–5 mg of both preparations reproducibly induced a 2–3-fold increase in cytotoxicity at Day 3 (data not shown). Thus i.v. was more reliable than i.p. injection for augmentation of splenic cytotoxicity.

However, the i.v. injection of small doses of *C. parvum* has been reported to depress splenic cytotoxicity in mice (Ojo *et al.*, 1978*a*). Accordingly, comparison was made between the effect of 1 $\mu$g and 1 mg of Preparation C injected i.v. on splenic cytotoxicity. Spleen cells were tested for NK activity in the standard assay with W/FuG-1 target cells (Fig. 2a) and for activity against antibody-coated P815 cells in an assay for ADCC (Fig. 2b). Clearly, whilst 1 mg stimulated cytotoxicity against W/FuG-1 cells, 1 $\mu$g caused a depression in cytotoxicity which was maximal at 10 days and returned to control levels by Day 25 (Fig. 2a). Splenomegaly and enhanced total cytotoxicity were observed until Day 13 with 1 mg, whereas 1 $\mu$g had no effect on spleen-cell number (data not shown).

FIG. 2.—Effect of i.v. *C. parvum* on NK cells and ADCC. W/Fu rats received 1 mg or 1 µg of Preparation C i.v. and spleen cells were assayed for cytotoxicity at various times on (a) W/FuG-1 cells or (b), antibody-coated P815 cells.

Similarly, whilst ADCC was stimulated by 1 mg at Day 3 it was depressed by 1 µg, as observed using W/FuG-1 target cells (Fig. 2b).

*Effect of T-cell depletion on cytotoxicity augmented by* C. parvum

T cells were depleted either by adult thymectomy, irradiation and marrow reconstitution (ATX.BM) rats (Table II),

TABLE II.—*Augmentation of cytotoxicity of PEC of normal and T-cell-depleted W/Fu rats**

| Group | Dose of *C. parvum* (mg) | CU (±s.e.) | Index of CU | TCU |
|---|---|---|---|---|
| Normal | — | 15·4±0·7 | 1·0 | 430 |
| Normal | 0·5 | 43·3±3·1 | 2·8 | 3020 |
| ATX.BM | — | 22·2±0·9 | 1·0 | 530 |
| ATX.BM | 0·5 | 53·6±4·1 | 2·4 | 1770 |

* Age-matched normal and ATX.BM rats received 0·5 mg of Preparation B *C. parvum* i.p. PEC were harvested 5 days later and assayed against W/Fu-G-1 target cells.

or by the use of a specific heterologous anti-T-cell antiserum and complement (Fig. 3).

Firstly, as previously observed (Shellam, 1977), ATX.BM rats exhibit naturally occurring cytotoxicity towards W/FuG-1 target cells (Table II). A similar pattern of response of PEC to *C. parvum* was seen

FIG. 3.—Effect of anti-T-cell serum on *C. parvum*—augmented cytotoxicity in the peritoneal exudate and spleen. (□) untreated cells and (⊡) antiserum- and complement-treated cells. Panels (a), (b): PEC from control (a), or *C. parvum*-injected (b) W/Fu rats receiving 0·5 mg of Preparation B i.p. 5 days before assay. Panels (c) (d): spleen cells from control (c) or *C. parvum*-injected (d) WAG rats receiving 1 mg of Preparation C i.v. 2 days before assay.

in normal and ATX.BM rats, although it should be noted that the total cytotoxicity was lower in ATX.BM than in intact rats after stimulation, and that this reflects the smaller increase in leucocyte numbers in these rats. Thus T cells do not appear to be essential for the augmentation of cytotoxic PEC by *C. parvum*

Secondly, cytotoxic cells from the peritoneal cavity or spleen of normal or *C. parvum*-stimulated rats were compared for their susceptibility to lysis by a specific anti-T-cell antiserum and complement (Fig. 3). The mean recovery of cells after this treatment was 50% for PEC and 40% for spleen cells. Treatment with antiserum or complement alone did not diminish cytotoxicity (data not shown). Cytotoxicity was enriched by the removal of T cells and even allowing for the loss of about half the cell population after treatment with antiserum and complement, it can be seen that the cytotoxicity in the spleen and peritoneal exudate of normal or boosted rats is largely mediated by non-T cells.

*Adherence properties of the cytotoxic cells*

The effect of the removal of adherent cells on the cytotoxicity of normal and *C. parvum*-boosted PEC and spleen cells was investigated by incubation of the cells in plastic Petri dishes (Table III). Most of the cytotoxicity was associated with the non-adherent population in both normal and *C. parvum*-boosted PEC or spleen cells. In all groups, adherent cells displayed modest cytotoxicity on a cell-for-cell basis, except the adherent cells from *C. parvum*-boosted peritoneal exudate (Table III). However, adherent cells were few in number and their contribution to total cytotoxicity was always a relatively minor one. Since the non-adherent cells were <5% positive for nonspecific esterase and contain <1% phagocytic cells (see Materials and Methods) the cytotoxic cells from *C. parvum*-stimulated rats resemble the NK cells of normal rats in being non-adherent and non-phagocytic and lacking nonspecific esterase.

TABLE III.—*Cytotoxicity in the adherent† and non-adherent fractions of spleen and PEC in normal and* C. parvum-*injected rats**

| Exp./Group | % Cell recovery | CU (±s.e.) | TCU recovered |
|---|---|---|---|
| I. Spleen control | | | |
| Unfractionated | 100 | 7·5±0·6 | 750 |
| Nonadherent | 54·8 | 10·6±1·5 | 580 |
| Adherent | 28·8 | 4·7±0·8 | 140 |
| Spleen *C. parvum* | | | |
| Unfractionated | 100 | 14·0±0·4 | 1400 |
| Nonadherent | 66·2 | 32·9±1·4 | 2180 |
| Adherent | 23·7 | 5·3±0·1 | 130 |
| II. PEC control | | | |
| Unfractionated | 100 | 30·6±2·3 | 3060 |
| Nonadherent | 41·7 | 32·4±2·6 | 1350 |
| Adherent | 21·3 | 4·8±0·6 | 100 |
| PEC *C. parvum* | | | |
| Unfractionated | 100 | 59·7±2·5 | 5970 |
| Non-adherent | 65·6 | 77·4±3·9 | 5080 |
| Adherent | 27·7 | 35·5±3·0 | 980 |

* Rats were injected i.p. with 0·5 mg of Preparation B of *C. parvum*, i.p. 5 days before assay. In Experiments I and II, W/Fu and WAG rats were used respectively.

W/FuG-1 target cells were used.

† Adherent cells were removed by incubation in plastic Petri dishes and recovered with lignocaine (see Materials and Methods).

*Specificity of cytotoxicity after treatment with* C. parvum

The cytotoxic specificity of spleen cells or PEC from normal and *C. parvum*-inoculated rats was compared in competitive inhibition assays, using target cells which were susceptible or resistant to NK-cell-mediated lysis (Fig. 4).

Clearly the specificity of cytotoxic cells in the spleen or peritoneal exudate of *C. parvum*-stimulated rats closely resembled that of the corresponding cells from normal rats, since a similar pattern of inhibition by a panel of unlabelled target cells was observed.

*Strain variation in response to* C. parvum

By specificity analysis using direct lysis, the spleen cells of i.v.-inoculated W/Fu rats closely resemble those of normal rats, lysing W/FuG-1 but not P815 or RBL-5 target cells (Table IV), and similar results were obtained with WAG rats (data not shown). In contrast, BN spleen cells expressed a different specificity after *C. parvum* inoculation i.v. Thus whilst the levels of NK cells in the spleens of normal BN rats were lower than in W/Fu, as observed previously (Shellam & Hogg, 1977), the degree of stimulation of cytotoxicity towards W/FuG-1 cells by *C. parvum* was greater than in W/Fu, and marked lysis of P815 and RBL-5 cells was found. This altered pattern of lysis in BN rats after inoculation of *C. parvum* was verified in 4 matched experiments with W/Fu spleen cells.

### DISCUSSION

In this study *Corynebacterium parvum* has been shown to enhance the cyto-

FIG. 4.—Specificity of PEC and spleen from normal and *C. parvum*-injected rats as shown by competitive inhibition. W/Fu rats were injected i.p. with 0·5 mg of Preparation B of *C. parvum* 5 days before assay. W/FuG-1 was used as the $^{51}Cr$-labelled target and unlabelled competitors were used at the ratios shown. (a) Normal spleen. (b) *C. parvum*-augmented spleen. (c) Normal PEC. (d) *C. parvum*-augmented PEC. ----- represents the $\%^{51}Cr$ release in the absence of a competitor. (■) Thymus; (▲) (C58NT)D; (◆) P815; (▼) Raji; (●) W/FuG-1 and (○) K562.

TABLE IV.—*Strain variation in the response to i.v. injection of* C. parvum*

| | | Target cells | | | | | |
|---|---|---|---|---|---|---|---|
| | Strain of | W/FuG-1 | | P815 | | RBL-5 | |
| Group | rat | CU | TCU | CU | TCU | CU | TCU |
| Control | W/Fu | 29·0 ± 1·4 | 6100 | 1·8 ± 0·1 | 390 | 1·2 ± 0·1 | 260 |
| *C. parvum* | W/Fu | 40·7 ± 2·3 | 16190 | 4·3 ± 0·2 | 1700 | 2·8 ± 0·1 | 1100 |
| Control | BN | 13·7 ± 0·5 | 3140 | 2·8 ± 0·1 | 640 | 0·3 ± 0·1 | ND |
| *C. parvum* | BN | 60·4 ± 3·2 | 20410 | 26·0 ± 1·0 | 8780 | 14·3 ± 1·1 | 4830 |

* Preparation C of *C. parvum* was injected i.v. in a dose of 1 mg 2 days before assay. All groups assayed simultaneously.

ND Not done as CU values too low to give realistic estimate of TCU.

toxicity of peritoneal exudate and splenic cells towards W/FuG-1 lymphoma target cells in a short-term $^{51}Cr$-release assay. The cytotoxic cells have been shown to resemble the NK cells of normal rats. The effects of *C. parvum* were influenced by the route of inoculation, the dose, the time after injection at which the response was examined, and the strain and preparation of *C. parvum* which was used. In addition to its effects on cytotoxicity, *C. parvum* also enhanced leucocyte numbers in these organs, markedly increasing the total cytotoxic activity in each organ.

The i.p. inoculation of *C. parvum* augmented cytotoxicity in the peritoneal exudate, doses in the range 0·5–1 mg being the most effective, and doses greater than 1 mg being generally less stimulatory. On a body-weight-adjusted basis this dose response pattern is similar to that found with PEC in mice (Ojo *et al.*, 1978a). Interestingly, the 3 preparations of *C. parvum* had different abilities to augment cytotoxicity in the peritoneal exudate. Thus heat-killed organisms of the ATCC 11829 strain (Preparation A) were less effective than Strain CN6134 prepared similarly (Preparation C), and a formalin-killed preparation of CN6134 (Preparation B) seemed less effective than heat-killed organisms of the same strain. This last observation appears to be related to the method of preparation rather than to the presence of thiomersal preservative in Preparation B, since the i.p. injection of the same volume of thiomersal saline had no effect on cytotoxicity in the peritoneal cavity. Organisms of Strain CN6134 were clearly more potent than those of the ATCC 11829 strain, and produced more marked changes in the investigated parameters: increased cytotoxicity and leucocyte number in the peritoneal cavity and ability to augment splenic cytotoxicity after i.p. inoculation. Recent surveys have shown similar interstrain variations in the biological activities of *C. parvum* in mice (O'Neill *et al.*, 1973; McBride *et al.*, 1975) including anti-tumour activity and the ability to induce splenomegaly and inflammatory peritoneal exudates (McBride *et al.*, 1975).

Some differences have been found in the ability of *C. parvum* to augment splenic cytotoxicity after i.p. inoculation, ranging from stimulation (Herberman *et al.*, 1977) to no change (Ojo *et al.*, 1978*a*). Our results suggest that these differences are a property of the strain and preparation of *C. parvum* used, since Preparations A and B infrequently stimulated splenic cytotoxicity by this route, whilst Preparation C always did so. Indeed Preparations A and B occasionally depressed splenic cytotoxicity. Since cytotoxicity in the peritoneal exudate was always stimulated by this route of inoculation, the data suggest that augmentation of cytotoxicity in the peritoneal cavity occurs independently of the spleen. This is strengthened by the finding that such augmentation of cytotoxicity in *C. parvum*-inoculated splenectomized rats was equivalent to that in normal rats (Flexman & Shellam, unpublished observations).

In contrast, whilst i.p. inoculation only stimulated the spleen if a potent preparation of *C. parvum* was used, i.v. inoculation regularly augmented splenic cytotoxicity irrespective of the preparation, though the latter response was time- and dose-dependent. Thus 1 mg augmented cytotoxicity initially but the response rapidly declined to control levels. A dose of 1 $\mu$g, however, only depressed the cytotoxicity, but this was more apparent at later times. A similar response to the i.v. inoculation of *C. parvum* has been reported in mice (Ojo *et al.*, 1978*a*). ADCC to antibody-coated P815 cells was similarly depressed by 1 $\mu$g, though 1 mg was stimulatory. The similarity of the responses to high and low doses of *C. parvum* by NK cells and cells active in ADCC provides support for the concept that the cells involved in these 2 mechanisms are closely related (Ojo & Wigzell, 1978). These several effects of *C. parvum* may reflect different thresholds for the induction of certain responses, such as the production of interferon at high doses and the development of suppressor cells, or a mechanism which interferes with the maturation of NK cells at low doses. We have examined spleen-cell preparations from rats inoculated with 1 $\mu$g for the presence of suppressor cells, using them in cell mixtures with control or *C. parvum*-stimulated spleen cells, with no satisfactory evidence for their presence.

It was found that the *C. parvum*-augmented cytotoxic cells closely resembled NK cells in their physical properties and cytotoxic specificity. Thus cytotoxic cells from the spleen or peritoneal exudate of *C. parvum*-stimulated rats were non-adherent, non-phagocytic, negative for nonspecific esterase and non-T cells. In addition to the data on the plastic adherence of the effector cells from *C. parvum*-stimulated rats, in further experiments with nylon-wool columns most of the cytotoxicity recovered was in the non-adherent population (Flexman & Shellam, unpublished observation). This finding would seem to rule out a contribution to cytotoxicity by B cells. In addition it was shown that augmentation of cytotoxicity by *C. parvum* could occur in the absence of the thymus, since marked augmentation was found in ATX.BM rats. It was noted, however, that whilst the cytotoxic cells from *C. parvum*-treated rats were largely non-adherent, significant cytotoxicity was manifested by a subpopulation of PEC which adhered to plastic, but this population was less apparent in the peritoneal cavity of normal rats. Heterogeneity in adherence properties of cytotoxic cells in the peritoneal exudate has also been seen in rats whose cytotoxicity was boosted with tumour cells (Dawkins & Shellam, 1979*b*).

The cytotoxic specificity of cells from normal and *C. parvum*-inoculated rats, whether tested by competitive inhibition or by direct lysis, was found to be similar. Thus *C. parvum* resembles tumour cells (Dawkins & Shellam, 1979*b*) and Newcastle disease virus (Flexman & Shellam, unpublished observation) in augmenting cytotoxicity in rats which closely resembles that of NK cells in its selective lysis. Comparable observations have been made with a variety of agents in mice (Herberman *et al.*, 1977; Wolfe *et al.*, 1977; Ojo *et al.*, 1978*b*) and evidence suggests that the common property of such stimulatory agents is the ability to induce the formation of interferon (Gidlund *et al.*, 1978), the NK-stimulating effect of which has been well documented (Trinchieri & Santoli, 1978; Gidlund *et al.*, 1978; Djeu *et al.*, 1979).

Another interesting feature emerged from our specificity studies. Whereas splenic NK cells from normal W/Fu and BN rats had equivalent specificity, inoculation of *C. parvum* altered the specificity of cytotoxic cells from BN rats in such a way that there was lysis of P815 and RBL-5 target cells. In contrast, little lysis of these cells was effected by spleen cells from *C. parvum*-stimulated W/Fu rats, although cytotoxicity towards the sensitive W/FuG-1 target cell was increased. Strain differences in the specificity of NK cells towards peritoneal target cells have been reported in virus-infected mice (Welsh *et al.*, 1979). The explanation for strain differences in the specificity of NK cells is not known.

Whilst it has been shown that NK cells stimulated by *C. parvum* may be responsible for tumour regression (Ojo, 1979) the effect of *C. parvum* on other cell types with anti-tumour potential has been well documented (Ghaffar *et al.*, 1975) and tumour regression may thus result from the stimulation of several mechanisms. However, the present study has focused on the effect of *C. parvum* on NK cells in the rat, and has shown that cytotoxicity of these cells can be augmented, but under carefully selected conditions.

We are grateful to Karen Dewhirst and Mary Ann Chan for excellent technical assistance and to Professor N. F. Stanley for his support.

This study was supported by grants from the National Health and Medical Research Council of Australia and the Cancer Council of Western Australia.

REFERENCES

BASH, J. A. (1978) Suppression of rat T-cell proliferation by *Corynebacterium parvum*: T-cell requirements for induction. *J. Reticuloendothel. Soc.*, **23**, 63.

DAWKINS, H. J. S. & SHELLAM, G. R. (1979*a*) Augmentation of cell-mediated cytotoxicity to a rat lymphoma. I. Stimulation of non-T-cell cytotoxicity *in vivo* by tumour cells. *Int. J. Cancer*, **24**, 235.

DAWKINS, H. J. S. & SHELLAM, G. R. (1979*b*) Augmentation of cell-mediated cytotoxicity to a rat lymphoma. II. Characterization of the non-T cytotoxic cells stimulated *in vivo* by tumour cells as natural killer cells. *Int. J. Cancer*, **24**, 244.

DE LANDAZURI, M. O., KEDAR, E. & FAHEY, J. L. (1974) Synergistic co-operation between isoantiserum and immune lymphoid cells: *In vitro* studies with a syngeneic rat lymphoma. *J. Immunol.*, **112**, 2102.

DJEU, J. Y., HEINBAUGH, J. A., HOLDEN, H. T. & HERBERMAN, R. B. (1979) Augmentation of mouse natural killer cell activity by interferon and interferon inducers. *J. Immunol.*, **122**, 175.

GHAFFAR, A., CULLEN, R. T. & WOODRUFF, M. F. A. (1975) Further analysis of the anti-tumour effect *in vitro* of peritoneal exudate cells from mice treated with *Corynebacterium parvum*. *Br. J. Cancer*, **31**, 15.

GIDLUND, M., ÖRN, A., WIGZELL, H., SENIK, A. & GRESSER, I. (1978) Enhanced NK cell activity in mice injected with interferon and interferon inducers. *Nature*, **273**, 759.

HALLER, O., HANSSON, M., KIESSLING, R. & WIGZELL, H. (1977) Role of non-conventional natural killer cells in resistance against syngeneic tumour cells *in vivo*. *Nature*, **270**, 609.

HERBERMAN, R. B., NUNN, M. E., HOLDEN, H. T., STAAL, S. & DJEU, J. Y. (1977) Augmentation of natural cytotoxic reactivity of mouse lymphoid cells against syngeneic and allogeneic target cells. *Int. J. Cancer*, **19**, 555.

HERBERMAN, R. B., NUNN, M. E. & LAVRIN, D. H. (1975) Natural cytotoxic reactivity of mouse lymphoid cells against syngeneic and allogeneic tumours. I. Distribution of reactivity and specificity. *Int. J. Cancer*, **16**, 216.

KIRCHNER, H., GLASER, M. & HERBERMAN, R. B. (1975) Suppression of cell-mediated tumour immunity by *Corynebacterium parvum*. *Nature*, **257**, 396.

MCBRIDE, W. H., DAWES, J., DUNBAR, N., GHAFFAR, A. & WOODRUFF, M. F. A. (1975) A comparative study of anaerobic Coryneforms. Attempts to correlate their anti-tumour activity with their serological properties and ability to stimulate the lymphoreticular system. *Immunology*, **28**, 49.

MILAS, L. & SCOTT, M. T. (1978) Anti-tumour activity of *Corynebacterium parvum*. *Adv. Cancer Res.*, **26**, 257.

MÖLLER, G. & MÖLLER, E. (1976) The concept of immunological surveillance against neoplasia. *Transplant. Rev.*, **28**, 1.

OEHLER, J. R., LINDSAY, L. R., NUNN, M. E., HOLDEN, H. T. & HERBERMAN, R. B. (1978) Natural cell-mediated cytotoxicity in rats. II. *In vivo* augmentation of NK-cell activity. *Int. J. Cancer*, **21**, 210.

OJO, E. (1979) Positive correlation between the levels of natural killer cells and the *in vivo* resistance to syngeneic tumour transplants as influenced by various routes of administration of *Corynebacterium parvum* bacteria. *Cell. Immunol.*, **45**, 182.

OJO, E. & WIGZELL, H. (1978) Natural killer cells may be the only cells in normal mouse lymphoid cell populations endowed with cytotoxic ability for antibody-coated tumour target cells. *Scand. J. Immunol.*, **7**, 297.

OJO, E., HALLER, O., KIMURA, A. & WIGZELL, H. (1978*a*) An analysis of conditions allowing *Corynebacterium parvum* to cause either augmentation or inhibition of natural killer cell activity against tumour cells in mice. *Int. J. Cancer*, **21**, 444.

OJO, E., HALLER, O. & WIGZELL, H. (1978*b*) *Corynebacterium parvum*-induced peritoneal exudate cells with rapid cytotoxic activity against tumour cells are non-phagocytic cells with characteristics of natural killer cells. *Scand. J. Immunol.*, **8**, 215.

O'NEILL, G. J., HENDERSON, D. C. & WHITE, R. G. (1973) The role of anaerobic Coryneforms on specific and non-specific immunological reactions. I. Effect on particle clearance and humoral and cell-mediated immunological responses. *Immunology*, **24**, 977.

SCOTT, M. T. (1975) Potentiation of the tumor-specific immune response by *Corynebacterium parvum*. *J. Natl Cancer Inst.*, **55**, 65.

SHELLAM, G. R. (1974) Studies on a Gross-virus induced lymphoma in the rat. I. The cell-mediated immune response. *Int. J. Cancer*, **14**, 65.

SHELLAM, G. R. (1977) Gross-virus-induced lymphoma in the rat. V. Natural cytotoxic cells are non-T-cells. *Int. J. Cancer*, **19**, 225.

SHELLAM, G. R. & HOGG, N. (1977) Gross-virus-induced lymphoma in the rat. IV. Cytotoxic cells in normal rats. *Int. J. Cancer*, **19**, 212.

STUART, A. E., HEBESHAW, J. A. & DAVIDSON, E. A. (1978) Phagocytes *in vitro*. In *Handbook of Experimental Immunology*. Ed. Weir. Oxford: Blackwell, 3rd edn, **2**, 31.

STUTMAN, O. (1975) Immunodepression and malignancy. *Adv. Cancer Res.*, **22**, 261.

TRINCHIERI, G. & SANTOLI, D. (1978) Anti-viral activity induced by culturing lymphocytes with tumour-derived or virus-transformed cells. Enhancement of human natural killer cell activity by interferon and antagonistic inhibition of susceptibility of target cells to lysis. *J. Exp. Med.*, **147**, 1314.

WELSH, R. M., ZINKERNAGEL, R. M. & HALLENBECK, L. A. (1979) Cytotoxic cells induced during lymphocytic choriomeningitis virus infection of mice. II. Specificities of the natural killer cells. *J. Immunol.*, **122**, 475.

WOLFE, S., TRACEY, D. E. & HENNEY, C. S. (1977) BCG-induced murine effector cells. II. Characterization of natural killer cells in peritoneal exudates. *J. Immunol.*, **119**, 1152.

WOODRUFF, M. F. A., DUNBAR, N. & GHAFFAR, A. (1973) The growth of tumours in T-cell deprived mice and their response to treatment with *Corynebacterium parvum*. *Proc. R. Soc. Lond. B.*, **184**, 97.

WOODRUFF, M. F. A. & WARNER, N. L. (1977) Effect of *Corynebacterium parvum* on tumor growth in normal and athymic (nude) mice. *J. Natl Cancer Inst.*, **58**, 111.